# Supplementary material for: Multifaceted interventions to decrease mortality in patients with severe sepsis/septic shock—a quality improvement project
Source: PeerJ. 2015 Oct 20;3:e1290. doi: 10.7717/peerj.1290 (PMC4614979; doi:10.7717/peerj.1290)
Supplement: Supplemental Information 1 [file peerj-03-1290-s001.pdf]

Date	ICU_Admit	ICU_Discha	Age	Gender (M	PatientHei	PatientWei	BMI
########							
########	########	########	83.445	1	155	40.8	16.98231
########	########	########	76.082	1	160	60.6	23.67188
########	########	########	88.889	1	154	62.1	26.18485
########	########	########	81.04	0	157	90	36.51264
########	########	########	88.711	0	168	71.1	25.19133
########	########	########	63.314	1	160	58.6	22.89063
########	########	########	63.962	1	154	77.5	32.67836
########	########	########	76.038	1	165	70	25.71166
########	########	########	78.456	1	179	86.8	27.09029
########	########	########	60.667	1	168	94.9	33.62387
########	########	########	74.82	0	170	92.3	31.93772
########	########	########	75.891	1	163	75.9	28.56713
########	########	########	84.829	0	181	74.8	22.83203
########	########	########	59.628	0	180	104.3	32.19136
########	########	########	90.136	1	160	62.6	24.45313
########	########	########	48.747	0	194	104	27.63312
########	########	########	50.007	0	187	87.5	25.02216
########	########	########	62.837	0	168	88	31.17914
########	########	########	72.727	0	178	103.8	32.76102
########	########	########	70.283	0	164	110	40.89827
########	########	########	63.94	1	147	107.7	49.84034
########	########	########	66.3	1	146	63	29.55526
########	########	########	56.046	0	182	126.9	38.31059
########	########	########	78.13	0	182	78.9	23.81959
########	########	########	54.804	1	199	100.4	25.3529
########	########	########	61.253	0	180	126.6	39.07407
########	########	########	62.861	1	165	83.7	30.7438
########	########	########	75.034	0	170	49.7	17.19723
########	########	########	68.104	0	182	84.9	25.63096
########	########	########	59.596	0	172	65.4	22.10654
########	########	########	52.544	1	167	53.2	19.07562
########	########	########	81.116	0	157	90	36.51264
########	########	########	63.324	0	183	67.7	20.21559
########	########	########	73.1	1	156	92.1	37.84517
########	########	########	88.322	0	164	58.1	21.60173
########	########	########	56.27	0	178	106	33.45537
########	########	########	53.73	1	159	75.5	29.86432
########	########	########	58.971	1	175	123.9	40.45714
########	########	########	50.45	0	187	99.5	28.45377
########	########	########	80.077	0	188	95.2	26.93526
########	########	########	58.546	0	168	68.3	24.19926
########	########	########	75.787	1	160	69	26.95313
########	########	########	63.461	0	183	117	34.93684
########	########	########	80.93	1	147.3	55	25.34879
########	########	########	31.904	0			

########	########	########	84.189	0	175	109.2	35.65714
########	########	########	58.089	0	168	161.8	57.3271
########	########	########	45.39	1	127	89.9	55.73811
########	########	########	37.842	0	186	87.3	25.23413
########	########	########	56.935	0	189	198.9	55.68153
########	########	########	75.099	0	172	84.5	28.56274
########	########	########	50.189	1	177	79.9	25.50353
########	########	########	51.479	0	187	125.8	35.97472
########	########	########	89.912	1	160	74.4	29.0625
########	########	########	62.307	0	193	90.9	24.40334
########	########	########	59.553	1	149	78.2	35.22364
########	########	########	82.427	1	150	66.6	29.6
########	########	########	60.348	0	168		
########	########	########	66.762	1	177	124.6	39.77146
########	########	########	76.342	1	162	63.7	24.27221
########	########	########	64.775	1	155	101.4	42.20604
########	########	########	88.027	1	157	80.9	32.8208
########	########	########	70.449	0	165	55.3	20.31221
########	########	########	71.216	1	152	63	27.26801
########	########	########	93.844	0	162	72.2	27.51105
########	########	########	71.901	1	165	95	34.8944
########	########	########	54.745	0	176	97.6	31.50826
########	########	########	64.966	1	155	64.5	26.84703
########	########	########	89.439	1	170		
########	########	########	106.523	0	160	69.9	27.30469
########	########	########	85.269	0	177.8	88.1	27.86842
########	########	########	80.326	0	164	102.2	37.99822
########	########	########	52.518	0	180	72.1	22.25309
########	########	########	80.698	1	172	49.6	16.76582
########	########	########	38.598	1			
########	########	########	79.493	0	182.9	98.8	29.53449
########	########	########	86.153	0	170	90.3	31.24567
########	########	########	64.898	1	155	48.3	20.10406
########	########	########	61.211	1			
########	########	########	64.156	1	156		
########	########	########	54.055	1			
########	########	########	53.092	1	165		
########	########	########	79.762	0			
########	########	########	86.805	1			
########	########	########	24.854	1	138		
########	########	########	78.537	1	154		
########	########	########	69.989	0	183		
########	########	########	60.208	1			
########	########	########	67.142	0			
########	########		43	0			

Admission_	Admitting_	ServiceCod	ICU_Lengtl	ICU_Discha	ICU_Discha	Hospital_A	Hospital_D	Hospital_Le
MB6B and	DIR_ADMI1	CCS	3.37	DO5D	0	########	########	24.04
MB6B and	DO3D	CCS	3.02	JO3G	0	########	########	5.86
MB6B and	DO6D	CCS	1.08	HOSP DC	1	########	########	1.67
MB6B and	ETU	CCS	1.22	DO6D	0	########	########	7.1
MB6B and	DO3D	CCS	2.35	HOSP DC	1	########	########	9.6
MB6B and	DIR_ADMI1	CCS	2.03	HOSP DC	1	########	########	2.03
MB6B and	ETU	CCS	2.08	HOSP DC	0	########	########	2.08
MB6B and	ETU	CCS	2.53	DO6D	0	########	########	23.44
MB6B and	DO3D	CCS	0.77	DO3D	0	########	########	3.51
MB6B and	ETU	CCS	2.43	DO6D	0	########	########	3.46
MB6B and	ETU	CCS	1.93	JO3B	0	########	########	14.02
MB6B and	DIR_ADMI1	CCS	1.85	DO5D	0	########	########	6.84
MB6B and	DIR_ADMI1	CCS	6.25	HOSP DC	1	########	########	6.25
MB6B and	ETU	CCS	1.67	DO3D	0	########	########	7.76
MB6B and	DIR_ADMI1	CCS	3.79	DO6B	0	########	########	28.02
MB6B and	MB3C	CCS	1.04	DO6B	0	########	########	6
MB6B and	ETU	CCS	0.52	DO6B	0	########	########	3.36
MB6B and	MB5E	CCS	0.9	MB5E	0	########	########	17.27
MB6B and	ETU	CCS	1.32	DO3D	0	########	########	10.4
MB6B and	ETU	CCS	4.27	JO3B	0	########	########	11.06
MB6B and	ETU	CCS	7.76	MB6F	0	########	########	19.71
MB6B and	ETU	CCS	0.52	DO5D	0	########	########	3.58
MB6B and	DIR_ADMI1	CCS	1.74	DO3D	0	########	########	3.87
MB6B and	ETU	CCS	3.19	JO3B	0	########	########	4.06
MB6B and	DIR_ADMI1	CCS	3.95	DO6D	0	########	########	13.93
MB6B and	MB6F	CCS	1.35	MB6F	0	########	########	31.56
MB6B and	ETU	CCS	15.42	JO3G	0	########	########	37.2
MB6B and	DO6B	CCS	0.83	JO3B	0	########	########	2.58
MB6B and	ETU	CCS	0.65	JO3B	0	########	########	1.7
MB6B and	ETU	CCS	2.03	DO3D	0	########	########	3
MB6B and	ETU	CCS	1.68	JO3B	0	########	########	6.76
MB6B and	ETU	CCS	1.01	JO3B	0	########	########	9.15
MB6B and	ETU	CCS	6.18	HOSP DC	1	########	########	6.18
MB6B and	ETU	CCS	6.83	JO3G	0	########	########	8.77
MB6B and	DIR_ADMI1	CCS	2.16	DO6D	0	########	########	18.73
MB6B and	DIR_ADMI1	CCS	18.18	JO3G	0	########	########	19.7
MB6B and	DIR_ADMI1	CCS	6.05	DO3D	0	########	########	8.32
MB6B and	ETU	CCS	1.78	JO3G	0	########	########	15.72
MB6B and	ETU	CCS	0.55	MB5D	0	########	########	2.57
MB6B and	ETU	CCS	1.99	DO3D	0	########	########	6.16
MB6B and	ETU	CCS	1.78	JO3G	0	########	########	4.05
MB6B and	DIR_ADMI1	CCS	5.32	MB5E	0	########	########	9.19
MB6B and	ETU	CCS	1.77	DO5D	0	########	########	3.88
MB6B and	ETU	CCS	3.79	HOSP DC	1	########	########	3.79
MB6B and	ETU	CCS	3.58	JO3B	0	########	########	15.85

MB6B and	ETU	CCS	2.01	JO3G	0	########	########	8.07
MB6B and	DO6D	CCS	10.41	HOSP DC	1	########	########	16.82
MB6B and	ETU	CCS	3.18	DO5D	0	########	########	6.14
MB6B and	ETU	CCS	1.99	ANES	0	########	########	68.85
MB6B and	FR5C	CCS	14.66	MB6F	0	########	########	27.8
MB6B and	DIR_ADMIT	CCS	5.54	DO6B	0	########	########	9.64
MB6B and	ETU	CCS	19.31	DO6B	0	########		
MB6B and	ETU	CCS	2.56	DO3D	0	########	########	5.63
MB6B and	DIR_ADMIT	CCS	10.32	MB6F	0	########	########	26.17
MB6B and	ETU	CCS	1.46	JO3G	0	########	########	3.66
MB6B and	ETU	CCS	0.76	JO3G	0	########	########	2.81
MB6B and	ETU	CCS	3.22	JO3B	0	########	########	9.12
MB6B and	DIR_ADMIT	CCS	5.83	HOSP DC	1	########	########	5.83
MB6B and	DIR_ADMIT	CCS	6.04	JO3B	0	########	########	14.04
MB6B and	DIR_ADMIT	CCS	0.92	DO6D	0	########	########	5.11
MB6B and	ETU	CCS	1.44	DO6D	0	########	########	5.54
MB6B and	ETU	CCS	0.99	JO3B	0	########	########	2.88
MB6B and	DIR_ADMIT	CCS	2.04	JO3G	0	########	########	5.8
MB6B and	MB7G	CCS	2.05	MB5D	0	########	########	26.79
MB6B and	ETU	CCS	1.28	DO3D	0	########	########	3.05
MB6B and	DIR_ADMIT	CCS	1.08	DO3D	0	########	########	3.17
MB6B and	DIR_ADMIT	CCS	1.73	JO3B	0	########	########	3.78
MB6B and	ETU	CCS	1.7	DO5D	0	########	########	9.61
MB6B and	ETU	CCS	0.79	MB4D	0	########	########	11.48
MB6B and	ETU	CCS	9.91		0	########		
MB6B and	ETU	CCS	1.46	DO2D	0	########	########	4.19
MB6B and	ETU	CCS	0.58	JO3G	0	########	########	3.46
MB6B and MB6G					2	########		
MB6B and	ETU	CCS	0.4	MB7G	0	########	########	1.06
MB7B and	FR1C	GEN	0.92	FR1C	0	########	########	5.21
MB6B and	JO3G	CCS	3.71		0	########		
MB6B and	DIR_ADMIT				2	########		
MB6B and	ETU	CCS	1.55		0	########		
E103 and E	PACU	ORT	1.8	E082	0	########	########	8.18
MB6B and	DIR_ADMIT	CCS	2.71	DO5D	0	########	########	17.55
MB6B and	ETU	MED	1.17	DO6B	0	########	########	2.03
MB6B and	JO3B	CCS	2.42	JO3B	0	########	########	7.43
MB6B and	ETU	CCS	0.38	DO2D	0	########	########	6.28
MB6B and	ETU	CCS	1.71	DO5D	0	########	########	4.49
MB6B and	DIR_ADMIT				2	########		
MB6B and MB6G		CCS	1.4	JO3G	0	########	########	3.63
MB6B and	ETU	FAM	12.24	DO2D	0	########	########	18.83
MB6B and	DO6D	CCS	0.21	ANES	0	########	########	33.79
MB8D and	PACU	NES	0.68	HOSP DC	0	########	########	1.03
MB6B and	ER		0.63	FLOOR	0	########	########	0.64

Hospital_D	Hospital_D	Apache3_S	Invasive_V	Invasive_V	Noninvasiv	Noninvasiv	Vent_Days	SOFA_day1
LTERM CAF	0	73	0	0	0	0		6
DEATH	1	110	0	0	0	0		11
DEATH	1	123	0	0	1	0.718727	0.718727	13
HOME	0	55	0	0	0	0		2
DEATH	1	98	0	0	0	0		9
DEATH	1	81	0	0	1	1.885417	1.885417	4
ACUTE CAR	0	107	0	0	1	0.197917	0.197917	10
HOME	0	66	0	0	0	0		2
LTERM CAF	0	57	0	0	0	0		1
HOME	0	47	0	0	0	0		3
HOME	0	103	0	0	0	0		7
HOME	0	113	0	0	0	0		8
DEATH	1	86	1	1.885417	1	1.34375	3.229167	4
LTERM CAF	0	78	0	0	0	0		7
DEATH	1	117	1	2.916667	0	0	2.916667	10
REHAB	0	68	0	0	0	0		2
HOME	0	88	1	0.197917	0	0	0.197917	4
HOME	0	64	0	0	0	0		4
HOME	0	70	0	0	1	0.260417	0.260417	6
LTERM CAF	0	83	1	2.213715	1	0.138194	2.35191	9
LTERM CAF	0	73	1	1.236111	1	3.652778	4.888889	6
HOME	0	55	0	0	0	0		3
HOME	0	62	0	0	0	0		8
HOME	0	96	0	0	1	0.3	0.3	14
LTERM CAF	0	123	1	1.802083	0	0	1.802083	13
ACUTE CAR	0	111	1	1.34375	0	0	1.34375	12
LTERM CAF	0	158	1	6.062477	1	2.40625	8.468727	16
HOME	0	59	0	0	0	0		3
HOME	0	70	0	0	0	0		7
HOME	0	44	1	0.642188	0	0	0.642188	6
HOME	0	55	0	0	0	0		4
HOME	0	64	0	0	0	0		2
DEATH	1	88	0	0	1	1.052083	1.052083	4
HOME	0	113	1	4.78125	1	0.125	4.90625	9
DEATH	1	94	1	0.947917	0	0	0.947917	13
DEATH	1	127	1	13.41664	1	0.229167	13.64581	12
PSYCH	0	110	1	3.822917	1	1.270833	5.09375	15
LTERM CAF	0	57	0	0	1	0.427083	0.427083	3
HOME	0	39	0	0	0	0		2
LTERM CAF	0	85	0	0	0	0		6
HOME	0	61	0	0	0	0		7
LTERM CAF	0	84	0	0	0	0		8
HOME	0	76	0	0	0	0		5
DEATH	1	114	1	2.677083	0	0	2.677083	12
HOME	0	53	0	0	0	0		9

LTERM CAF	0	93	0	0	1	1.010417	1.010417	7
DEATH	1	148	1	6.926366	0	0	6.926366	17
LTERM CAF	0	98	1	1.614583	1	0.390162	2.004745	11
REHAB	0	106	1	1.927083	0	0	1.927083	13
LTERM CAF	0	77	1	8.592361	1	4.520833	13.11319	8
	0	97	1	4.21875	1	0.197917	4.416667	11
	2	69	1	14.96873	1	0.229167	15.19789	12
	0	92	0	0	0	0		9
	0	109	1	5.298611	1	3.052083	8.350694	12
	0	40	0	0	0	0		4
	0	77	1	0.475694	0	0	0.475694	7
	0	111	1	2.988866	0	0	2.988866	13
DEATH	1	108	1	4.531227	1	0.395833	4.92706	10
	0	69	1	4.947917	0	0	4.947917	13
	0	52	0	0	0	0		5
	0	56	0	0	0	0		8
	0	65	0	0	0	0		2
	0	63	1	0.941667	1	0.493866	1.435532	8
DEATH	1	71	0	0	0	0		6
	0	64	0	0	0	0		7
	0	43	0	0	0	0		4
	0	87	1	0.729167	0	0	0.729167	13
	0	67	0	0	0	0		7
	0	82	1	0.472222	0	0	0.472222	10
	2	118	1	7.138843	1	0.21875	7.357593	15
	0	73	0	0	0	0		4
	0	85	0	0	0	0		6
	2	58						
DEATH	1	118	1	0.291644	0	0	0.291644	17
HOME	0	41	0	0	0	0		0
	2	94	0	0	0	0		11
	2	160						
	2	69	0	0	1	0.489583	0.489583	6
LTERM CAF	0	60	0	0	1	1.354514	1.354514	3
	0	69	1	1.010394	0	0	1.010394	10
HOME	0	69	0	0	0	0		5
	0	62	0	0	0	0		8
LTERM CAF	0	71	0	0	0	0		1
LTERM CAF	0	174	0	0	0	0		4
	2	42						
	0	57	0	0	0	0		5
	0	60	1	8.15625	0	0	8.15625	9
	0	71	0	0	0	0		7
	0	75	0	0	0	0		1
HOME	0	99						

DEATH (No	LFU_DATE (unreliable)
0	
0	########
1	########
1	########
0	########
1	########
1	########
0	########
1	########
1	########
0	########
0	########
0	########
1	########
0	########
1	########
0	########
0	########
0	########
0	########
1	########
0	########
0	########
0	########
1	########
0	########
0	########
1	########
0	########
0	########
0	########
0	########
0	########
1	########
0	########
1	########
1	########
0	########
0	########
0	########
1	########
0	########
0	########
0	########
1	########
2	########

0	########
1	########
0	########
0	########
1	########
0	########
0	########
2	
2	
0	########
0	########
2	
1	
2	
2	
0	########
2	
0	########
2	
0	########
2	
0	########
0	########
0	
0	########
0	########
2	
2	
2	
0	
0	########
2	
0	########
0	########
0	
0	########
0	
0	########
0	########
2	
0	
0	
0	
0	
0	########
